# Supplementary material for: An integrated framework for building trustworthy data-driven epidemiological models: Application to the COVID-19 outbreak in New York City
Source: PLoS Comput Biol. 2021 Sep 8;17(9):e1009334. doi: 10.1371/journal.pcbi.1009334 (PMC8452065; doi:10.1371/journal.pcbi.1009334)
Supplement: S1 Text — (PDF) [file pcbi.1009334.s001.pdf]

S1 Text. Model development. We adopt the following step-by-step procedure to build our model:

1. Choose components of the dynamics to simulate the transmission of the disease: susceptible (S), exposed (E), presymptomatic (P), symptomatic (I), asymptomatic (A), hospitalized (H), isolated (Q), deceased (D), and recovered (R).

2. Identify the flow between the components and link them with directed arrows. Assign the rate of the flow with one parameter per arrow except for $S \to E$, which is defined by the transmission of the disease. See S1 Fig.

3. Change parameters such that every parameter has physical meaning. We use nine parameters (p, q, δ, d_E, d_P, d_I, d_A, d_H, d_Q) to replace ($w_1, \cdots, w_9$). See Eq. (1) for the formulas. Note that there is a one-to-one correspondence between the nine parameters we use and ($w_1, \cdots, w_9$).

4. Add vaccination dynamics $S \to R$.

$$\begin{cases} d_E = w_1 \\ d_P = w_2 + w_3 \\ \delta = \dfrac{w_2}{w_2 + w_3} \\ d_I = w_4 + w_5 \\ p = \dfrac{w_4}{w_4 + w_5} \\ d_A = w_6 \\ d_H = w_7 + w_8 \\ q = \dfrac{w_7}{w_7 + w_8} \\ d_Q = w_9 \end{cases} \iff \begin{cases} w_1 = d_E \\ w_2 = \delta d_P \\ w_3 = (1-\delta) d_P \\ w_4 = p d_I \\ w_5 = (1-p) d_I \\ w_6 = d_A \\ w_7 = q d_H \\ w_8 = (1-q) d_H \\ w_9 = d_Q \end{cases} \tag{1}$$
