# Supplementary material for: An integrated framework for building trustworthy data-driven epidemiological models: Application to the COVID-19 outbreak in New York City
Source: PLoS Comput Biol. 2021 Sep 8;17(9):e1009334. doi: 10.1371/journal.pcbi.1009334 (PMC8452065; doi:10.1371/journal.pcbi.1009334)
Supplement: S2 Text — (PDF) [file pcbi.1009334.s002.pdf]

S2 Text. Parameter settings. Parameter values for the fixed parameters are summarized in Table 2 in the main text. The percentage of infected people who never show the disease's symptoms is extracted from the CDC's current best estimate for this value [1]. The parameter δ is obtained by averaging the lower and upper bounds of the estimates for this value from different sources:

$$\delta = \frac{0.3 + 0.9}{2} = 0.6.$$

We use data from the hospitalization surveillance network used by the CDC to estimate the median number of days an individual spends hospitalized due to the disease [2]. The numbers provided are specific to individuals admitted to the ICU and not admitted to the ICU divided into age groups; therefore, we perform a weighted average of those values considering the demographic composition of NYC. According to S1 Table, the average number of days of hospitalization for individuals aged 18-49 years is:

$$3 \times (1 - 0.238) + 11 \times 0.238 = 4.904;$$

average number of days of hospitalization for individuals aged 50-64 years:

$$4 \times 0.639 + 14 \times 0.361 = 7.610;$$

average number of days of hospitalization for individuals aged ≥ 65 years:

$$6 \times 0.647 + 12 \times 0.353 = 8.118.$$

As a result, the total average number of days of hospitalization d_H for all age groups can be obtained in the following way:

$$d_H \approx \frac{4.904 + 7.610 + 8.118}{3} \approx 6.9.$$

References

1. CDC. COVID-19 Pandemic Planning Scenarios; 2020. https://www.cdc.gov/coronavirus/2019-ncov/hcp/planning-scenarios.html(10-July-2020).

2. CDC. Coronavirus Disease 2019 (COVID-19)–Associated Hospitalization Surveillance Network (COVID–NET); 2020. https://www.cdc.gov/coronavirus/9472019-ncov/covid-data/covid-net/purpose-methods.html(28-Aug-2020).
