# Supplementary material for: An integrated framework for building trustworthy data-driven epidemiological models: Application to the COVID-19 outbreak in New York City
Source: PLoS Comput Biol. 2021 Sep 8;17(9):e1009334. doi: 10.1371/journal.pcbi.1009334 (PMC8452065; doi:10.1371/journal.pcbi.1009334)
Supplement: S3 Text — (PDF) [file pcbi.1009334.s003.pdf]

S3 Text. Basic and control reproduction number of the model in Fig 3 in the main text. The reproduction number in Eq. (5) in the main text is obtained using the next generation matrix operator described in [1]. The vector of infected state variables without considering hospitalization is denoted by $\mathbf{x} = (E, P, I, A)$. We define $F = \left[\frac{\partial \mathscr{F}_i(x_0)}{\partial x_j}\right]$ and $V = \left[\frac{\partial \mathscr{V}_i(x_0)}{\partial x_j}\right]$, where $\mathscr{F}_i$ is the rate of appearance of new infections in the ith compartment; $\mathscr{V}_i = \mathscr{V}_i^- - \mathscr{V}_i^+$, where $\mathscr{V}_i^+$ is the rate of transfer of individuals into the ith compartment by all other means except for infection, and $\mathscr{V}_i^-$ is the rate of transfer of individuals out of the ith compartment; and

$$x_0 = (S := 1, E := 0, P := 0, I := 0, A := 0, H := 0, Q := 0, D := 0, R := 0)$$

is the disease–free equilibrium state of the system. We have

$$F = \begin{pmatrix} 0 & \beta\epsilon & \beta & \beta\epsilon \\ 0 & 0 & 0 & 0 \\ 0 & 0 & 0 & 0 \\ 0 & 0 & 0 & 0 \end{pmatrix} \quad \text{and} \quad V = \begin{pmatrix} d_E & 0 & 0 & 0 \\ -d_E & d_P & 0 & 0 \\ 0 & -d_P\delta & d_I & 0 \\ 0 & -d_P(1-\delta) & 0 & d_A \end{pmatrix}.$$

Then,

$$FV^{-1} = \begin{pmatrix} \beta\left(\frac{\epsilon(d_A - \delta d_P + d_P)}{d_A d_P} + \frac{\delta}{d_I}\right) & \beta\left(\frac{\epsilon(d_A - \delta d_P + d_P)}{d_A d_P} + \frac{\delta}{d_I}\right) & \frac{\beta}{d_I} & \frac{\beta\epsilon}{d_A} \\ 0 & 0 & 0 & 0 \\ 0 & 0 & 0 & 0 \\ 0 & 0 & 0 & 0 \end{pmatrix}.$$

Let $\rho(FV^{-1})$ denote the dominant eigenvalue of FV^{-1}. The control reproduction number is given by $\mathcal{R}_c = \rho(FV^{-1})$.

References

1. Diekmann O, Heesterbeek JAP, Metz JAJ. On the definition and the computation of the basic reproduction ratio R0 in models for infectious diseases in heterogeneous populations. Journal of Mathematical Biology. 1990; 28(4):365–382. doi:10.1007/BF00178324.
