# Supplementary material for: An integrated framework for building trustworthy data-driven epidemiological models: Application to the COVID-19 outbreak in New York City
Source: PLoS Comput Biol. 2021 Sep 8;17(9):e1009334. doi: 10.1371/journal.pcbi.1009334 (PMC8452065; doi:10.1371/journal.pcbi.1009334)
Supplement: S4 Text — (PDF) [file pcbi.1009334.s004.pdf]

S4 Text. Definition and algorithm for identifiability analysis.

Structural identifiability

Suppose we are given a dynamical system of the following abstract form

$$\begin{aligned} \frac{dX}{dt} &= f(X(t), \Theta, u(t)) \\ y(t) &= g(X(t), \Theta, u(t)), \end{aligned} \tag{1}$$

where $X = (X_1, \cdots, X_n)$ represents the state variables, $y = (y_1, \cdots, y_m)$ represents the observables, $\Theta = (\theta_1, \cdots, \theta_k)$ contains the parameters to identify, and $u(t)$ represents the input variable to the system. A parameter set Θ is called structurally identifiable if

$$g(X(t), \Theta, u(t)) = g(X(t), \Phi, u(t)) \implies \Theta = \Phi \tag{2}$$

for every $\Phi = (\phi_1, \cdots, \phi_k)$ in the same space as Θ. A single parameter $\theta_i \in \Theta$ is called structurally identifiable if

$$g(X(t), \Theta, u(t)) = g(X(t), \Phi, u(t)) \implies \theta_i = \phi_i. \tag{3}$$

The structural identifiability, defined above, is referred to as global identifiability in [1–4]. Global identifiability is stronger than the so-called local identifiability, which only requires Eq. (2) or Eq. (3) to hold in a neighbourhood $\mathcal{N}(\Theta)$ of Θ. Structural identifiability analysis is usually conducted before the fitting of the model and it does not rely on any data. We perform the structural identifiability analysis using the software SIAN [56], which is based on differential algebra and Taylor series expansion. Detailed documentations on the theory behind the software can be found in [5], and information regarding the algorithm can be obtained from [6]. We also checked structural identifiability using the software GenSSI 2.0 [7], which is based on the Lie derivatives of ODE models.

Practical identifiability and correlation matrix

A parameter is called practically identifiable if it can be uniquely determined from the discrete data points instead of the structure of the equation. A structurally identifiable parameter may not be practically identifiable given the noise and limited measure points in the data. Unlike structural identifiability, there is no consistent definition of practical identifiability in the literature. Nevertheless, several methods including correlation matrix methods have been proposed to describe practical identifiability quantitatively [8, 9].

Statistical correlation can be used to describe some of the practical non-identifiability phenomena. Specifically, one can use the Fisher Information Matrix (FIM) to calculate the correlation matrix of Θ in Eq. (1). FIM is defined as:

$$F = \sum_{i=1}^{T} S_i^T V^{-1} S_i, \tag{4}$$

where V is covariance matrix of the measurements error. $S_i = \frac{\partial \tilde{y}(t_i)}{\partial \Theta}$ is the sensitivity matrix at time t_i, which can be calculated by solving the adjoint equation:

$$\frac{dS_i}{dt} = \frac{\partial g(X(\cdot), \Theta)}{\partial X}(t_i)\, S_i + \frac{\partial g(X(\cdot), \Theta)}{\partial \Theta}(t_i) \tag{5}$$

using automatic differentiation libraries. It is proved that the correlation matrix is equal to F^{-1} by the Cramér–Rao theorem [10]. Once the correlation matrix is obtained, one

can determine whether a parameter is identifiable by checking whether it is correlated with the other parameters. If θ_i is strongly correlated with θ_j, then neither of these parameters is practically identifiable because they cannot be uniquely determined from the data.

References

1. Bellman R, Åström KJ. On structural identifiability. Mathematical Biosciences. 9131970;7(3-4):329–339.

2. Rothenberg TJ. Identification in parametric models. Econometrica: Journal of the Econometric Society. 1971; p. 577–591.

3. Glover K, Willems J. Parametrizations of linear dynamical systems: Canonical forms and identifiability. IEEE Transactions on Automatic Control.9181974; 19(6):640–646.

4. Thowsen A. Identifiability of dynamic systems. International Journal of Systems Science. 1978; 9(7):813–825.

5. Hong H, Ovchinnikov A, Pogudin G, Yap C. Global identifiability of differential models. Communications on Pure and Applied Mathematics. 2020; 73(9):1831–1879.

6. Hong H, Ovchinnikov A, Pogudin G, Yap C. SIAN: software for structural identifiability analysis of ODE models. Bioinformatics. 2019; 35(16):2873–2874.

7. Ligon TS, Fröhlich F, Chişş OT, Banga JR, Balsa-Canto E, Hasenauer J. GenSSI2.0: multi-experiment structural identifiability analysis of SBML models. Bioinformatics. 2018; 34(8):1421–1423.

8. Miao H, Xia X, Perelson AS, Wu H. On identifiability of nonlinear ODE models and applications in viral dynamics. SIAM review. 2011; 53(1):3–39.

9. Tuncer N, Le TT. Structural and practical identifiability analysis of outbreak models. Mathematical Biosciences. 2018; 299:1–18.

10. Rao CR. Information and the accuracy attainable in the estimation of statistical parameters. Reson J Sci Educ. 1945; 20:78–90.
