# Supplementary material for: An integrated framework for building trustworthy data-driven epidemiological models: Application to the COVID-19 outbreak in New York City
Source: PLoS Comput Biol. 2021 Sep 8;17(9):e1009334. doi: 10.1371/journal.pcbi.1009334 (PMC8452065; doi:10.1371/journal.pcbi.1009334)
Supplement: S5 Text — (PDF) [file pcbi.1009334.s005.pdf]

S5 Text. Definition and algorithm for model robustness. The procedure of Monte Carlo simulation for model robustness can be summarized into the following steps:

1. Solve Eq. (1) in S4 Text with Θ equal to the estimated value $\tilde{\Theta}$ to get the calibrated observables $\tilde{y}$ at the time stamps where the original data are sampled. For example, for NYC dataset in the fully-observed case, we assume $\tilde{y} = [I_{new}, H_{new}, D_{new}]$, i.e., the daily increase in I_{sum}, H_{sum}, and D_{sum}.

2. Multiply the calibrated observables $\tilde{y}_i$ by independent and identically distributed Gaussian random noise $\xi_i^{(j)} \sim \mathcal{N}(\mathbf{1}, \sigma^2 I_T)$, $1 \leq i \leq m$, $1 \leq j \leq M$, where T is the number of time stamps, I_T is the $T \times T$ identity matrix, and M is the number of Monte Carlo steps. By this method we generate a dataset of size M:

$$[Y^{(1)}, \cdots, Y^{(M)}] = [\tilde{y} \odot \xi^{(1)}, \cdots, \tilde{y} \odot \xi^{(M)}],$$

   where $\odot$ is the elementwise product and $\xi^{(j)} = [\xi_1^{(j)}, \cdots, \xi_m^{(j)}]$.

3. The parameters are estimated again using these perturbed observables $\{Y^{(j)}\}_{j=1}^{M}$. The estimated value for the ith unknown parameter θ_i using $Y^{(j)}$ is denoted as $\hat{\theta}_i^{(j)}$. The average relative error (ARE) for θ_i is defined as:

$$\text{ARE}(\theta_i) = \frac{1}{M\sigma} \sum_{j=1}^{M} \left| \frac{\hat{\theta}_i^{(j)} - \theta_i}{\theta_i} \right|.$$

   Finally, we define the maximum average relative error (MARE) of the model to be the largest ARE across all the model parameters:

$$\text{MARE} = \max_{i \in \{1, \cdots, k\}} \text{ARE}(\theta_i).$$

   If MARE < 1, we say that the model is robust to perturbation.
