# Supplementary material for: An integrated framework for building trustworthy data-driven epidemiological models: Application to the COVID-19 outbreak in New York City
Source: PLoS Comput Biol. 2021 Sep 8;17(9):e1009334. doi: 10.1371/journal.pcbi.1009334 (PMC8452065; doi:10.1371/journal.pcbi.1009334)
Supplement: S6 Text — (PDF) [file pcbi.1009334.s006.pdf]

S6 Text. Alternative setups of the model. The framework we propose is subject to assumptions used to construct the model. We have listed the central assumptions for model design and data acquisition in the paper. To explore some of those assumptions, we test the following setups: shifting the policy timeline at the beginning of the outbreak, assessing the model's versatility to include undetected infections, and considering alternate initial value from recently published literature.

- If we shift the policy stay-at-home order at the beginning of the outbreak from March 22 to March 17 in the fitting, we would get the results in S12 Fig. The fitting at the beginning of the pandemic does not match with the data as closely as the case when the actual date is used, but the fitting in the latter stages is not affected as much.

- The framework and model we propose can be modified to include undetected infections, though for the purpose of this study we focus only on the reported cases. The lack of reliable data and testing capacity at the beginning of the pandemic makes estimating undetected infections very challenging. Testing capacity increases over time in NYC, thus altering the portion of undetected infections over time. To evaluate the effect of undetected infections on our model, we can modify it in the following manner: asymptomatic individuals and undetected infections can be lumped into the same compartment and we can redefine the parameter δ as the proportion of ascertained symptomatic cases. Subramanian et al. estimated that the proportion of detected symptomatic cases is low at the beginning of the pandemic, ranging from 13% to 18%. We use the mean value $\delta = 0.155$ before March 22, 2020 and model a progressive linear increase in detection until June 8, 2020, after which we use constant $\delta = 0.6$. See S13 Fig for the parameter δ over time. We compare our original fitting in Fig 6 in the main text with the fitting with time-varying δ in S14 Fig.

- On top of the aforementioned time-dependent δ, we use the initial value of infected individuals from [1] to evaluate the effect on our model. The results are included in S15 Fig.

References

1. Gonzalez-Reiche AS, Hernandez MM, Sullivan MJ, Ciferri B, Alshammary H, Obla A, et al. Introductions and early spread of SARS-CoV-2 in the New York City area. Science. 2020; 369(6501):297–301.
