# Supplementary material for: An integrated framework for building trustworthy data-driven epidemiological models: Application to the COVID-19 outbreak in New York City
Source: PLoS Comput Biol. 2021 Sep 8;17(9):e1009334. doi: 10.1371/journal.pcbi.1009334 (PMC8452065; doi:10.1371/journal.pcbi.1009334)
Supplement: S1 Table — (PDF) [file pcbi.1009334.s007.pdf]

S1 Table. Data from CDC website (COVID-19 Pandemic Planning Scenarios) to estimate d_H.

	18-49 years	50-64 years	$\geq$ 65 years
Median number of days of hospitalization among those not admitted to ICU	3	4	6
Median number of days of hospitalization among those admitted to ICU	11	14	12
Percent admitted to ICU among those hospitalized	23.8%	36.1%	35.3%
