# Supplementary material for: An integrated framework for building trustworthy data-driven epidemiological models: Application to the COVID-19 outbreak in New York City
Source: PLoS Comput Biol. 2021 Sep 8;17(9):e1009334. doi: 10.1371/journal.pcbi.1009334 (PMC8452065; doi:10.1371/journal.pcbi.1009334)
Supplement: S2 Table — Different from Table 3, we assume H instead of Hsum is observed as data. (PDF) [file pcbi.1009334.s008.pdf]

S2 Table. Structural identifiability of the model with H as an observable. Structural identifiability is tested when H instead of H_{sum} is given as an observable. It turns out that the identifiability result is similar to Table 3.

Parameter	I_{sum}, H, D_{sum}	I_{sum}, H	H, D_{sum}	H
β	global	global	global	global
p	global	global	global	global
q	global	not	global	not
ϵ	global	global	global	global
δ	global	global	global	global
