# Supplementary material for: An integrated framework for building trustworthy data-driven epidemiological models: Application to the COVID-19 outbreak in New York City
Source: PLoS Comput Biol. 2021 Sep 8;17(9):e1009334. doi: 10.1371/journal.pcbi.1009334 (PMC8452065; doi:10.1371/journal.pcbi.1009334)
Supplement: S1 Fig — (PDF) [file pcbi.1009334.s009.pdf]

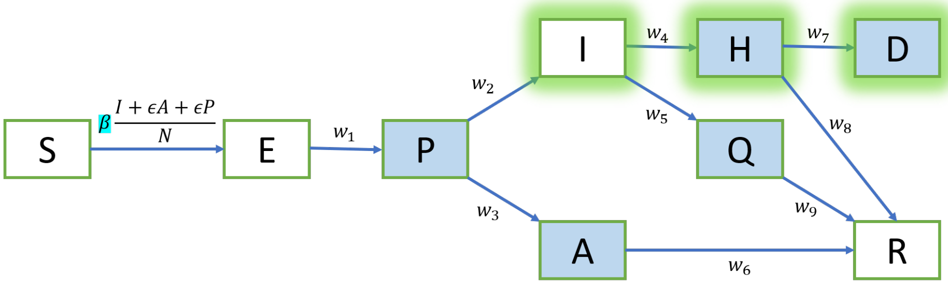

S1 Fig. Raw model. The graph demonstrates an intermediate step of building the proposed model in Fig 3 in the main text. It has the same compartmental structure as the final model but the inflows and outflows of different compartments are parameterized by unknown w_i's. It can be transformed to the final model through a change of variable in Eq. (1) in S1 Text and a supplement of vaccination dynamics.
