# Supplementary material for: An integrated framework for building trustworthy data-driven epidemiological models: Application to the COVID-19 outbreak in New York City
Source: PLoS Comput Biol. 2021 Sep 8;17(9):e1009334. doi: 10.1371/journal.pcbi.1009334 (PMC8452065; doi:10.1371/journal.pcbi.1009334)
Supplement: S2 Fig — (PDF) [file pcbi.1009334.s010.pdf]

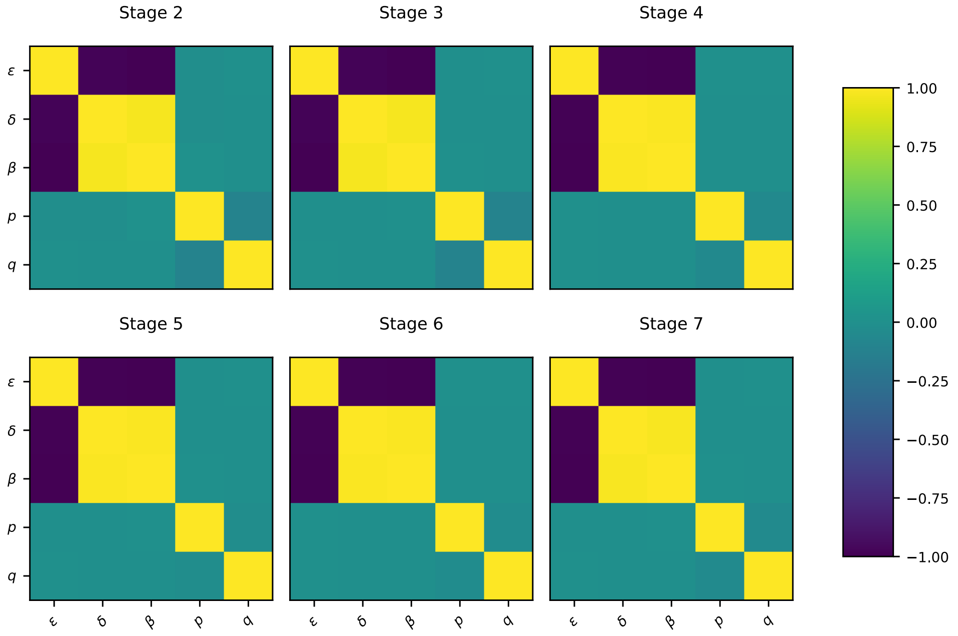

S2 Fig. Correlation matrix of β, p, q, δ, and ϵ in Stage 2 to Stage 7. Green means (almost) not statistically correlated while yellow/purple represents positively/negatively correlated. The correlation matrices are similar in theses stages, i.e., there is always correlation between β, ϵ, δ while there is no correlation between p, q and the other parameters.
