# Supplementary material for: An integrated framework for building trustworthy data-driven epidemiological models: Application to the COVID-19 outbreak in New York City
Source: PLoS Comput Biol. 2021 Sep 8;17(9):e1009334. doi: 10.1371/journal.pcbi.1009334 (PMC8452065; doi:10.1371/journal.pcbi.1009334)
Supplement: S3 Fig — (PDF) [file pcbi.1009334.s011.pdf]

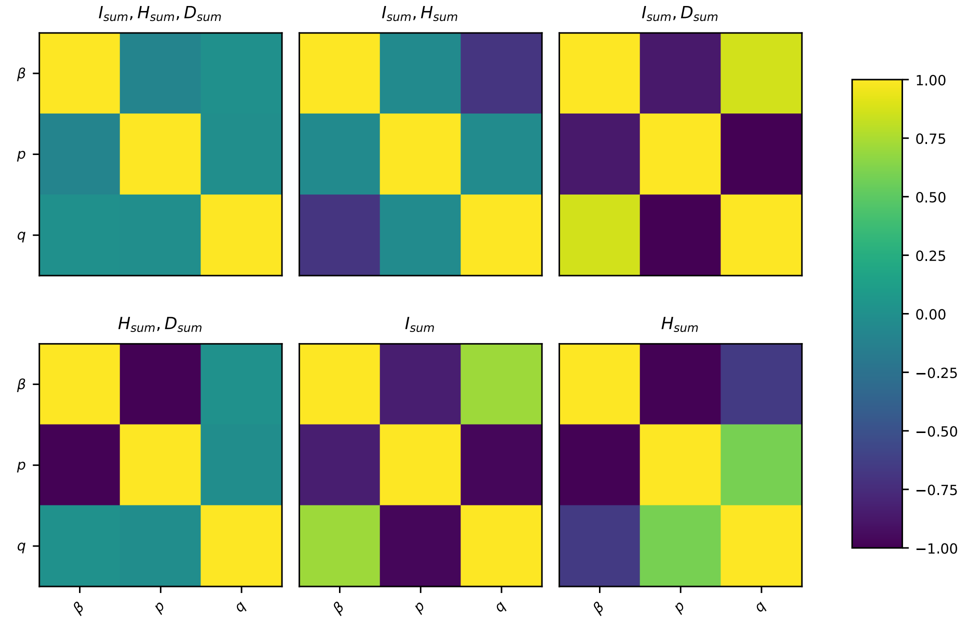

S3 Fig. The correlation matrix of β, p, and q in the setting of different observables, calculated in Stage 1. Green means (almost) not statistically correlated while yellow/purple represents positively/negatively correlated. When I_{sum}, H_{sum}, and D_{sum} are available, the model is practically identifiable. In other cases, there is correlation between the parameters.
