# Supplementary material for: An integrated framework for building trustworthy data-driven epidemiological models: Application to the COVID-19 outbreak in New York City
Source: PLoS Comput Biol. 2021 Sep 8;17(9):e1009334. doi: 10.1371/journal.pcbi.1009334 (PMC8452065; doi:10.1371/journal.pcbi.1009334)
Supplement: S4 Fig — (PDF) [file pcbi.1009334.s012.pdf]

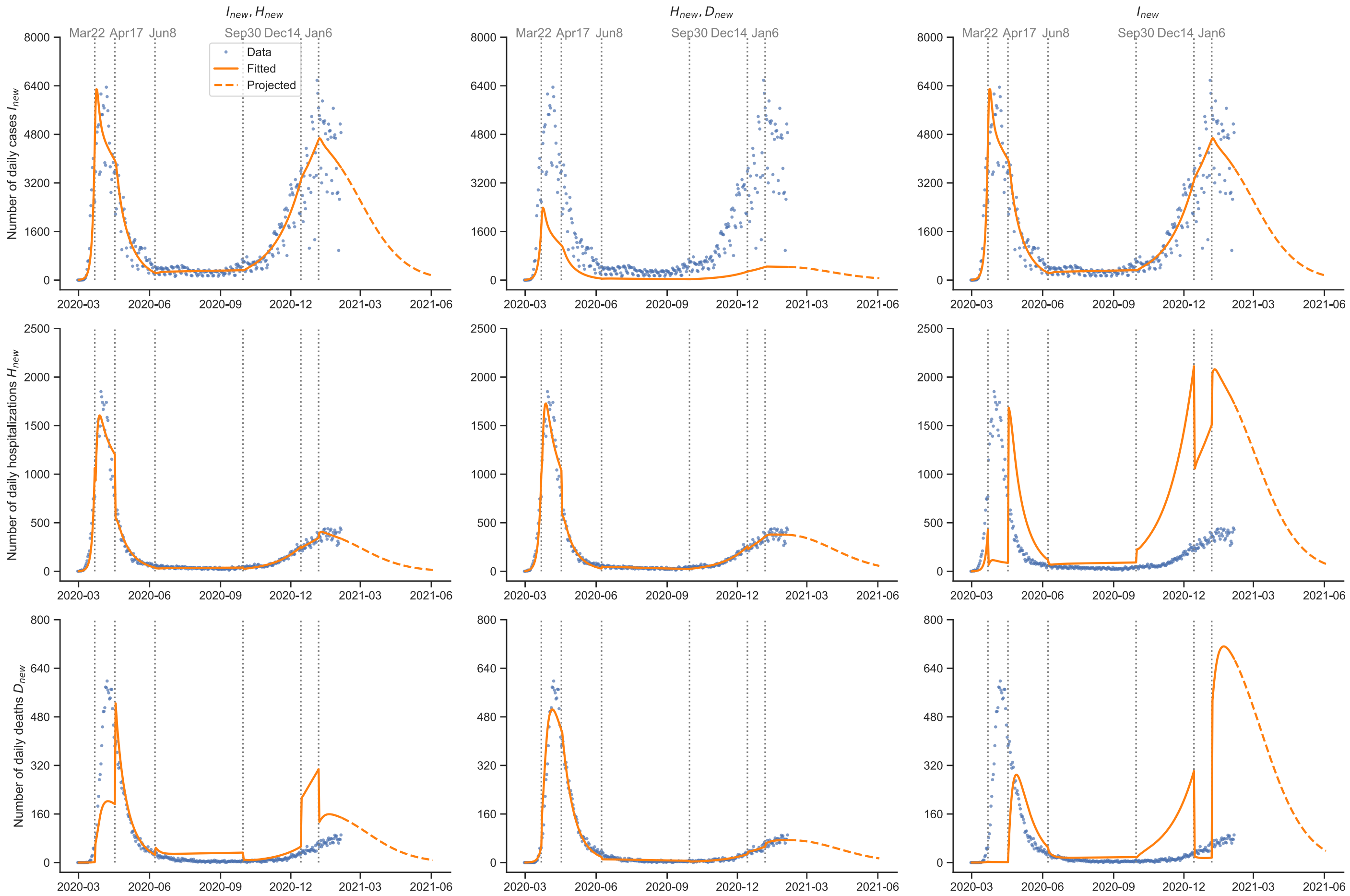

S4 Fig. Fitting and projection with the data given as (I_{new}, H_{new}), (H_{new}, D_{new}), or (I_{new}). Other settings are the same as Fig 6 in the main text. Each row represents the fitting and projection of an observable. Each column represents an observable setting. When only I_{new} and H_{new} are available, the fitting and projection for D_{new} is inaccurate, while the fitting and projection for I_{new} and H_{new} are correct. When only H_{new} and D_{new} are available, the fitting and projection for I_{new} is inaccurate, while the fitting and projection for H_{new} and D_{new} are correct. When only I_{new} is given, the model can only project I_{new} correctly.
