# Supplementary material for: An integrated framework for building trustworthy data-driven epidemiological models: Application to the COVID-19 outbreak in New York City
Source: PLoS Comput Biol. 2021 Sep 8;17(9):e1009334. doi: 10.1371/journal.pcbi.1009334 (PMC8452065; doi:10.1371/journal.pcbi.1009334)
Supplement: S5 Fig — (PDF) [file pcbi.1009334.s013.pdf]

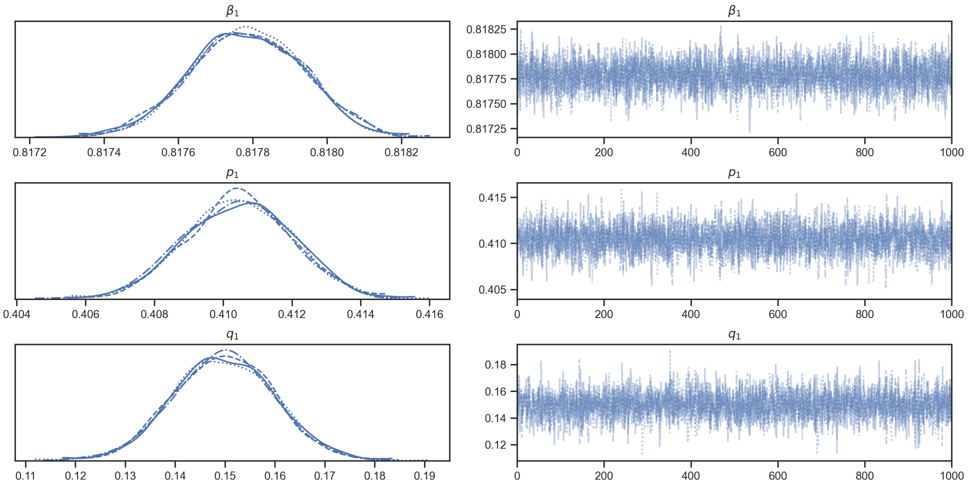

S5 Fig. MCMC simulation in Stage 1. Four chains of 1000 samples are drawn with 200 burn-in samples in every chain. [**Left Column**] Posterior distributions of the parameters (β, p, q). The narrow posterior distributions indicate that our numerical algorithm is robust, the quantity of data is sufficient for our approach of minimizing the loss function Eq. (14) in the main text, and the parameters are indeed close to constant. [**Right Column**] Sampling processes of the parameters. We can see that the chains are well-mixed, which implies the convergence of the sampling.
