# Supplementary figures and images for: An integrated framework for building trustworthy data-driven epidemiological models: Application to the COVID-19 outbreak in New York City
Source: PLoS Comput Biol. 2021 Sep 8;17(9):e1009334. doi: 10.1371/journal.pcbi.1009334 (PMC8452065; doi:10.1371/journal.pcbi.1009334)

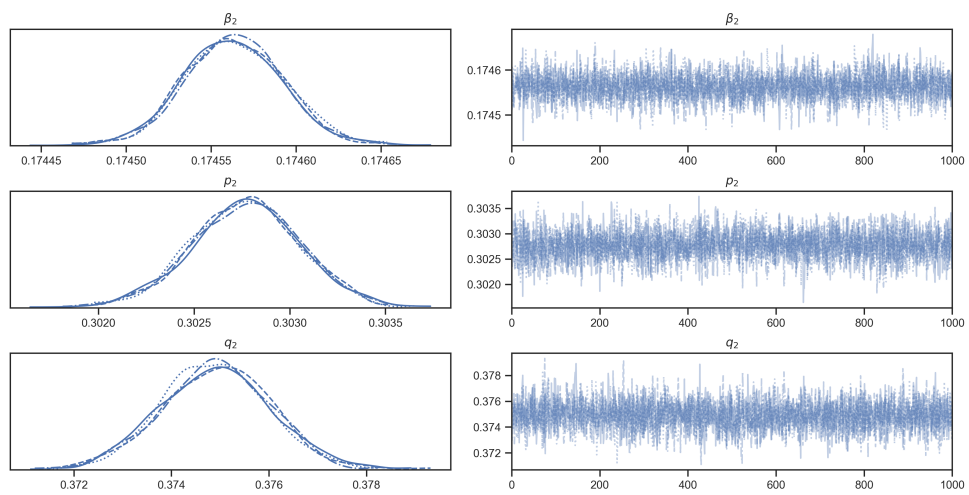

**S6 Fig.** MCMC simulation in Stage 2. Other settings are the same as S5 Fig.

Supplement: S6 Fig — (PDF) [file pcbi.1009334.s014.pdf]

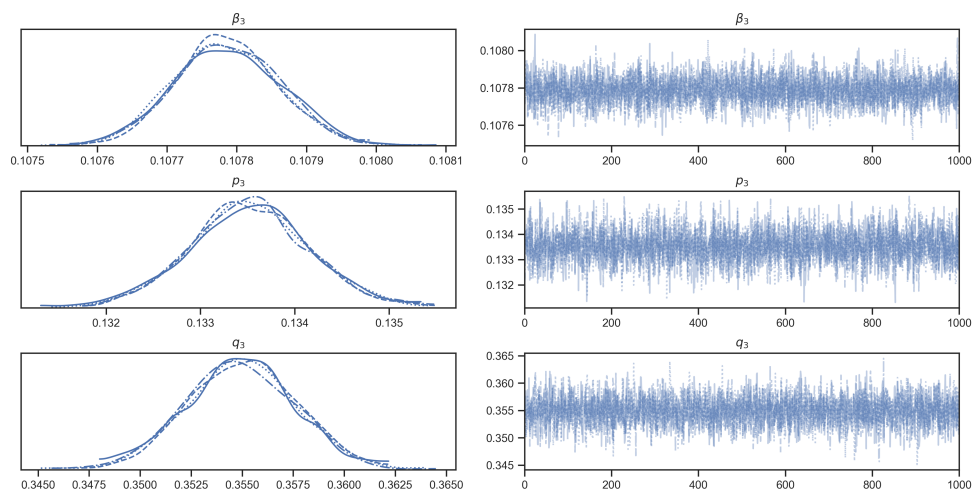

**S7 Fig.** MCMC simulation in Stage 3. Other settings are the same as S5 Fig.

Supplement: S7 Fig — (PDF) [file pcbi.1009334.s015.pdf]

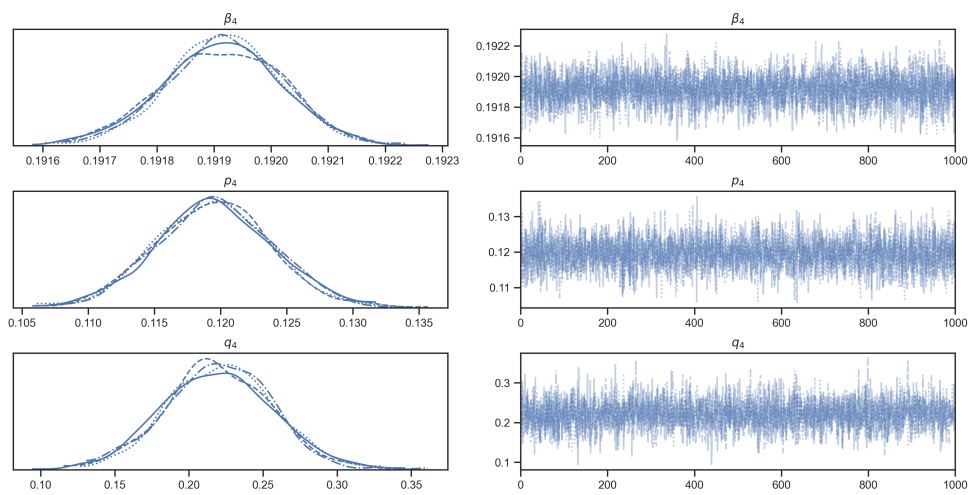

**S8 Fig.** MCMC simulation in Stage 4. Other settings are the same as S5 Fig.

Supplement: S8 Fig — (PDF) [file pcbi.1009334.s016.pdf]

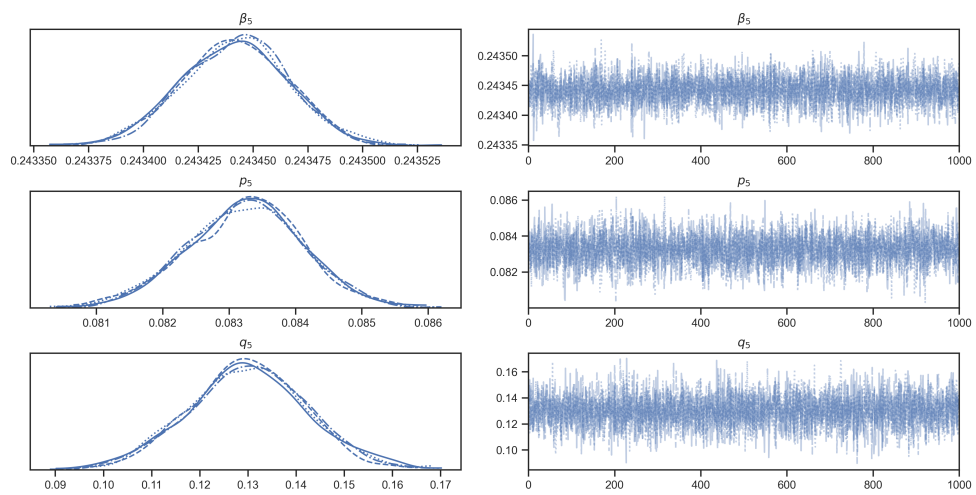

**S9 Fig.** MCMC simulation in Stage 5. Other settings are the same as S5 Fig.

Supplement: S9 Fig — (PDF) [file pcbi.1009334.s017.pdf]

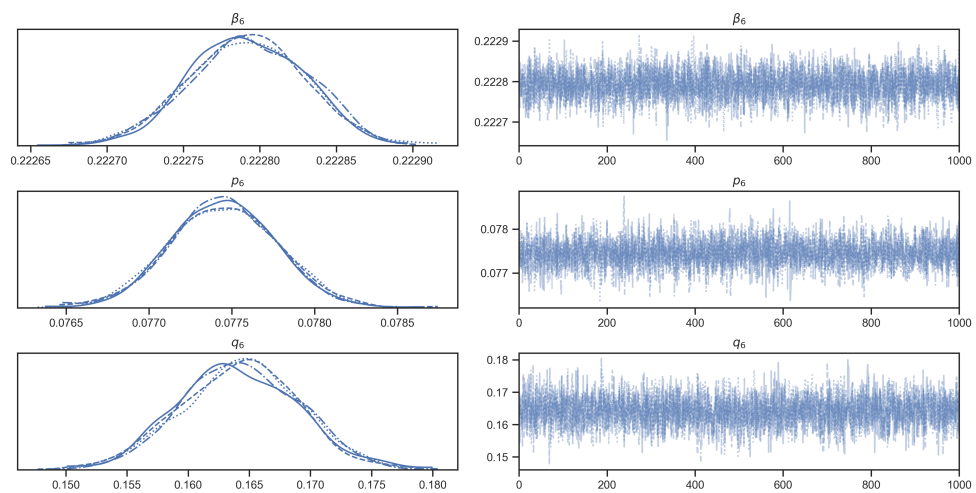

**S10 Fig. MCMC simulation in Stage 6.** Other settings are the same as S5 Fig.

Supplement: S10 Fig — (PDF) [file pcbi.1009334.s018.pdf]

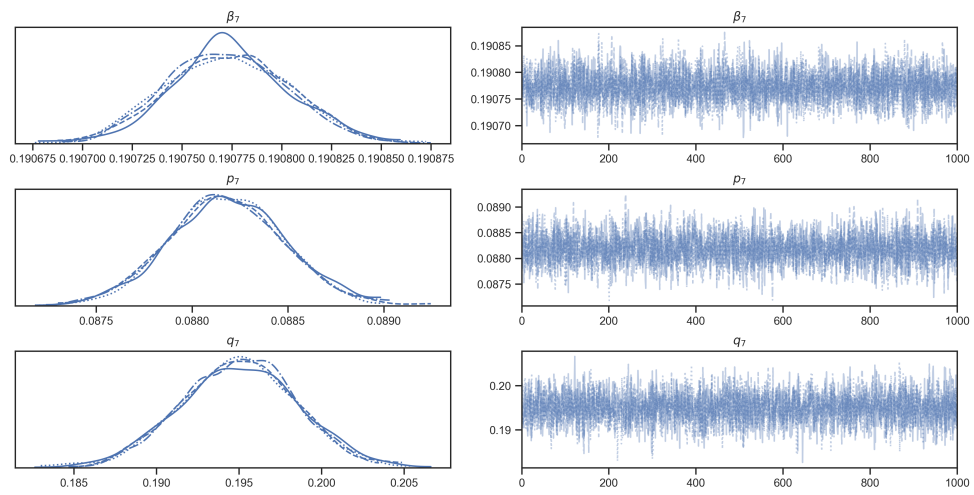

**S11 Fig. MCMC simulation in Stage 7.** Other settings are the same as S5 Fig.

Supplement: S11 Fig — (PDF) [file pcbi.1009334.s019.pdf]
