# Supplementary material for: An integrated framework for building trustworthy data-driven epidemiological models: Application to the COVID-19 outbreak in New York City
Source: PLoS Comput Biol. 2021 Sep 8;17(9):e1009334. doi: 10.1371/journal.pcbi.1009334 (PMC8452065; doi:10.1371/journal.pcbi.1009334)
Supplement: S12 Fig — (PDF) [file pcbi.1009334.s020.pdf]

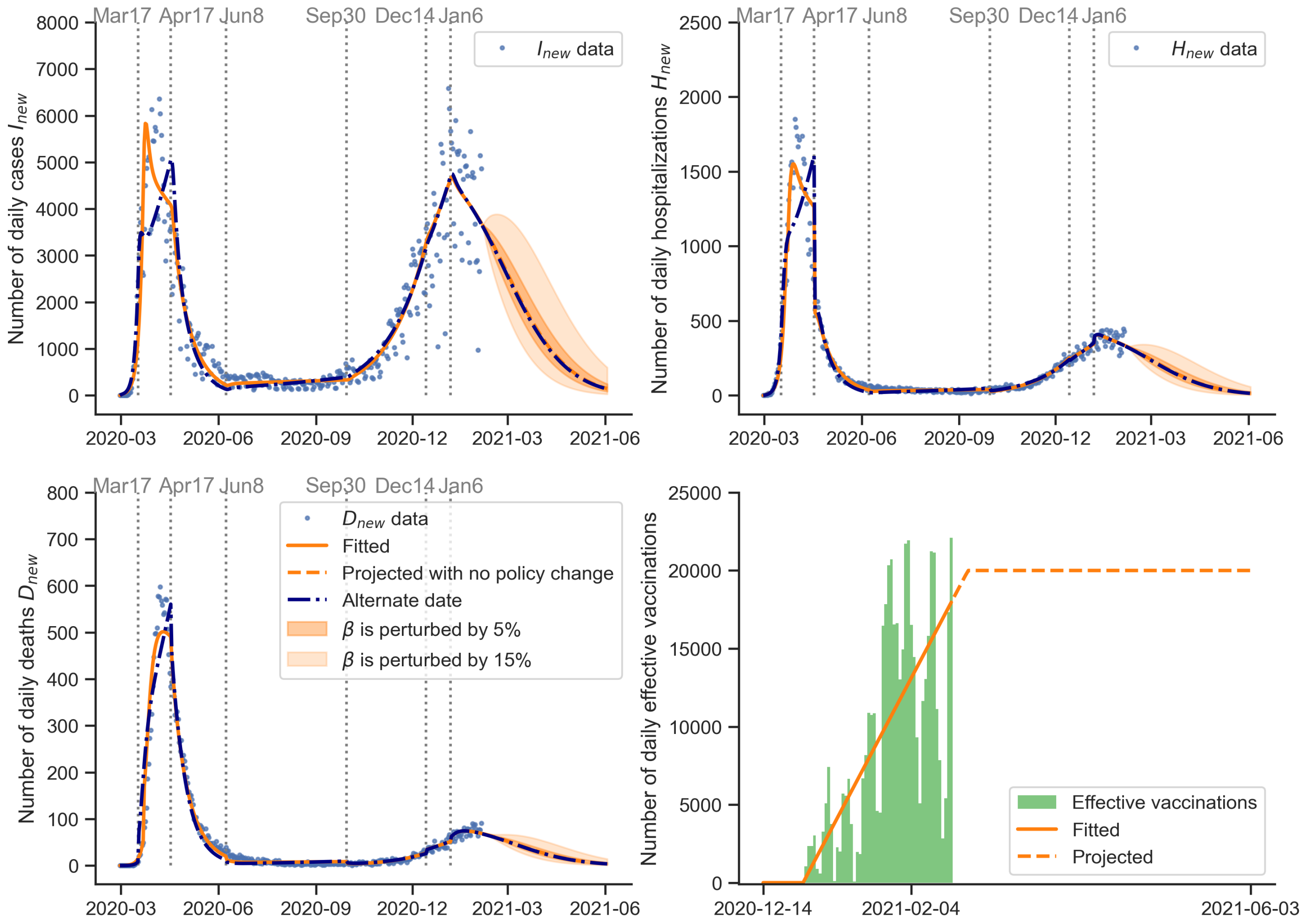

S12 Fig. Fitting and projection of $(I_{new}, H_{new}, D_{new})$. We overlay the fitting in Fig 6 in the main text (orange) with the dashed blue line fit if we shift the policy stay-at-home order at the beginning of the outbreak from March 22 to March 17. The fitting at the beginning of the pandemic does not match with the data as closely as the case when the actual date is used, but the fitting in the latter stages is not affected as much.
