# Supplementary material for: An integrated framework for building trustworthy data-driven epidemiological models: Application to the COVID-19 outbreak in New York City
Source: PLoS Comput Biol. 2021 Sep 8;17(9):e1009334. doi: 10.1371/journal.pcbi.1009334 (PMC8452065; doi:10.1371/journal.pcbi.1009334)
Supplement: S13 Fig — (PDF) [file pcbi.1009334.s021.pdf]

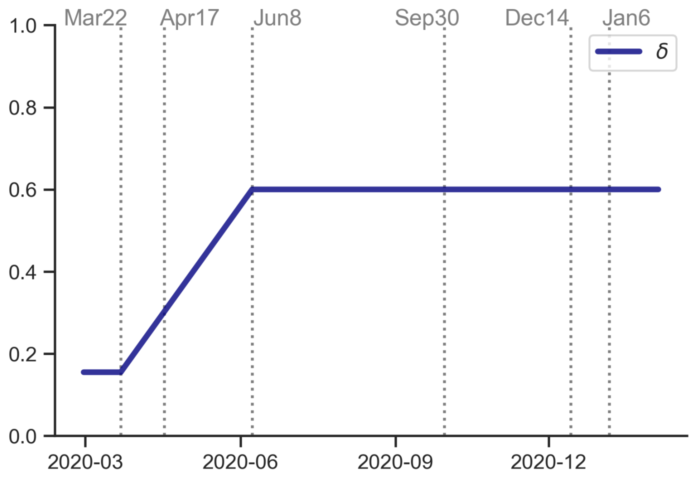

S13 Fig. Plot of the time-dependent ascertainment ratio δ. We use $\delta = 0.155$ before March 22, 2020 and model a progressive linear increase in detection until June 8, 2020, after which we use constant $\delta = 0.6$.
