# Supplementary material for: An integrated framework for building trustworthy data-driven epidemiological models: Application to the COVID-19 outbreak in New York City
Source: PLoS Comput Biol. 2021 Sep 8;17(9):e1009334. doi: 10.1371/journal.pcbi.1009334 (PMC8452065; doi:10.1371/journal.pcbi.1009334)
Supplement: S15 Fig — (PDF) [file pcbi.1009334.s023.pdf]

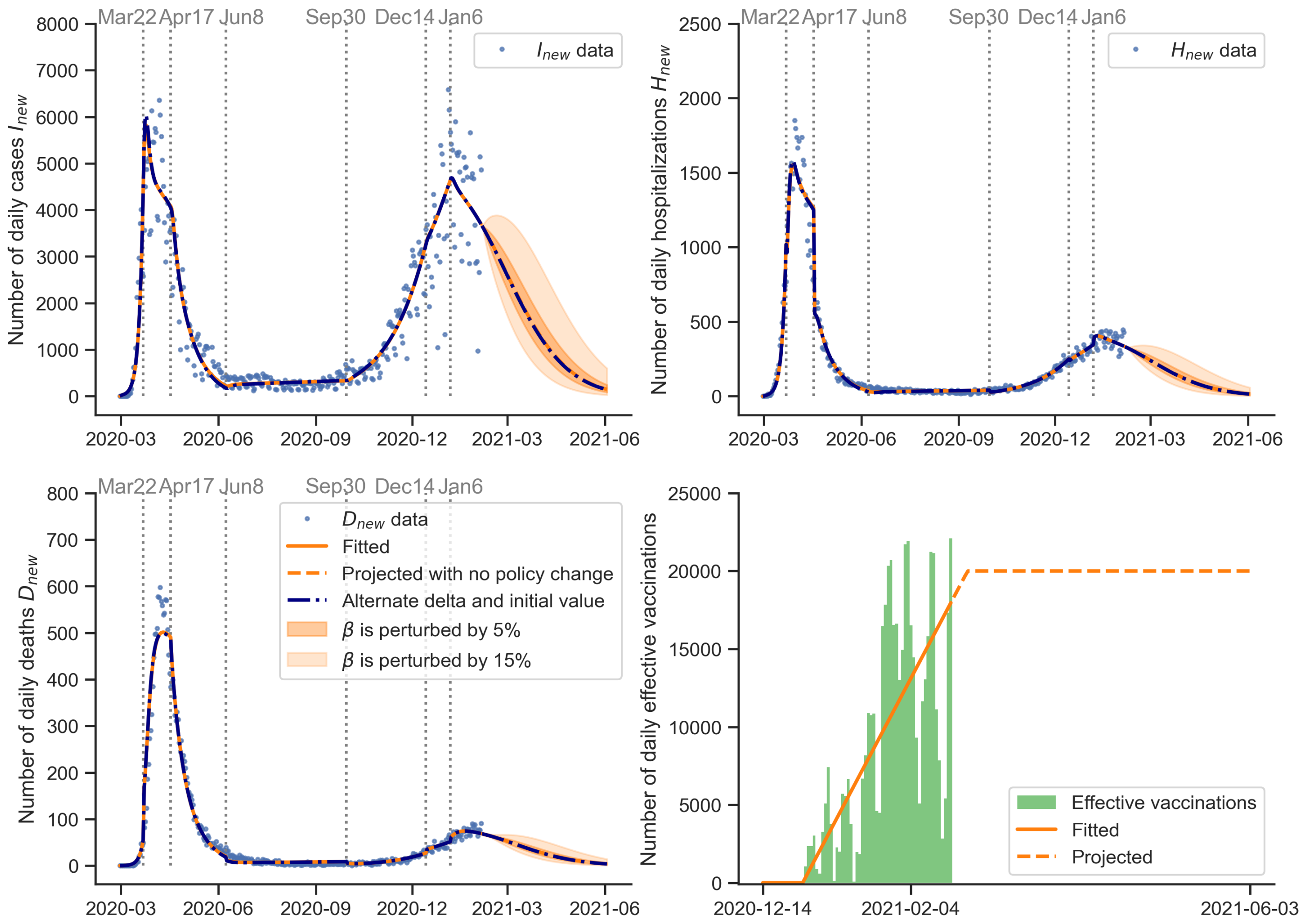

S15 Fig. Fitting and projection of $(I_{new}, H_{new}, D_{new})$ with time-dependent ascertainment ratio δ defined in S13 Fig and alternate initial value for infected and symptomatic individuals $I(0) = 4$. We overlay the fitting in Fig 6 in the main text (orange) with the dashed blue line fit, which considers a time-dependent δ and an alternate initial value.
